# *Salmonella* SPI1 Effector SipA Persists after Entry and Cooperates with a SPI2 Effector to Regulate Phagosome Maturation and Intracellular Replication

**DOI:** 10.1016/j.chom.2007.02.001

**Published:** 2007-03-15

**Authors:** Lyndsey C. Brawn, Richard D. Hayward, Vassilis Koronakis

**Affiliations:** 1 University of Cambridge, Department of Pathology, Tennis Court Road, Cambridge CB2 1QP, United Kingdom

**Keywords:** CELLBIO, MICROBIO

## Abstract

*Salmonellae* employ two type III secretion systems (T3SSs), SPI1 and SPI2, to deliver virulence effectors into mammalian cells. SPI1 effectors, including actin-binding SipA, trigger initial bacterial uptake, whereas SPI2 effectors promote subsequent replication within customized *Salmonella*-containing vacuoles (SCVs). SCVs sequester actin filaments and subvert microtubule-dependent motors to migrate to the perinuclear region. We demonstrate that SipA delivery continues after *Salmonella* internalization, with dosage being restricted by host-mediated degradation. SipA is exposed on the cytoplasmic face of the SCV, from where it stimulates bacterial replication in both nonphagocytic cells and macrophages. Although SipA is sufficient to target and redistribute late endosomes, during infection it cooperates with the SPI2 effector SifA to modulate SCV morphology and ensure perinuclear positioning. Our findings define an unexpected additional function for SipA postentry and reveal precise intracellular communication between effectors deployed by distinct T3SSs underlying SCV biogenesis.

## Introduction

Many microbes and parasites evade host immune responses by replicating in customized vacuoles within mammalian cells. Intracellular bacterial pathogens forge a specialized niche by delivering multiple virulence effectors into the cell that subvert trafficking events and alter vacuole positioning (Salcedo and Holden, 2005). Pathogen-containing vacuoles can be exploited as “Trojan horses” to track how intracellular compartments mature and migrate. Deciphering how bacterial effectors function provides not only critical understanding of infection but also new insights into endogenous membrane trafficking.

*Salmonellae* replicate in a modified phagosome termed the *Salmonella*-containing vacuole (SCV) in both nonphagocytic epithelial cells and macrophages (Knodler and Steele-Mortimer, 2003). SCVs interact transiently with early endosomes (EE), undergo Rab7- and phosphoinositide-dependent maturation (Brumell et al., 2001; Meresse et al., 1999), acidify (Rathman et al., 1996), and acquire markers characteristic of late endosomes (LE) and lysosomes (Lys), including lysosomal-associated membrane protein 1 (LAMP1) (Brumell et al., 2001). Replicative SCVs recruit actin filaments (F-actin) and hijack microtubule-dependent motors to migrate to the perinuclear region (Boucrot et al., 2005; Guignot et al., 2004; Harrison et al., 2003; Marsman et al., 2004; Meresse et al., 2001), where they intercept secretory traffic from the Golgi apparatus (GA) (Kuhle et al., 2006; Salcedo and Holden, 2003). Once positioned, maturation is stalled and bacterial replication is initiated. Specialized LAMP1-rich tubulovesicular structures of unknown function termed *Salmonella*-induced filaments (Sifs) extend along microtubules from the SCV (Stein et al., 1996).

*Salmonellae* encode two distinct T3SSs on chromosomal pathogenicity islands 1 (SPI1) and SPI2. Six SPI1 effectors coordinately trigger cytoskeletal rearrangements to force bacterial internalization into nonphagocytic cells (Hayward and Koronakis, 2002). Among these, *Salmonella* invasion protein A (SipA) binds actin and enhances entry efficiency by promoting actin polymerization and preventing filament disassembly (McGhie et al., 2001, 2004; Zhou et al., 1999). SPI2 effectors act subsequently in both epithelial cells and macrophages to promote intracellular replication and systemic spread (Galan, 2001). While the repertoire and activities of SPI2 effectors remain largely unknown, the majority localize to the cytoplasmic face of the SCV and often along Sifs (Henry et al., 2006; Knodler and Steele-Mortimer, 2005; Salcedo and Holden, 2003; Kuhle and Hensel, 2002).

Perhaps the best-characterized SPI2 effector is SifA, which is essential for Sif formation (Stein et al., 1996), SCV integrity, and *Salmonella* replication (Beuzon et al., 2000). Bacteria lacking SifA fail to commandeer the SifA-kinesin interacting protein (SKIP), a host kinesin inhibitor, allowing detrimental motor accumulation that triggers aberrant SCV migration toward the cell periphery (Boucrot et al., 2005). SifA has acquired a eukaryotic membrane-targeting motif and might also mimic host Rab GTPases (Alto et al., 2006; Boucrot et al., 2003).

The current tenet is that SPI1 and SPI2 effector cohorts function sequentially and autonomously, yet increasing evidence potentially challenges this view (Lawley et al., 2006; Hernandez et al., 2004; Knodler and Steele-Mortimer, 2003; Steele-Mortimer et al., 2002). Here we demonstrate that the SPI1 effector SipA continues to act from the cytosolic face of the SCV long after *Salmonella* entry. Not only can SipA independently induce LE redistribution, it also cooperates with the SPI2 effector SifA during infection to ensure perinuclear SCV positioning. The data reveal an essential contribution of a SPI1 effector to subsequent SCV maturation and bacterial replication and illuminate unanticipated intracellular communication between bacterial effectors deployed by distinct T3SSs.

## Results

### SipA Persists after Entry and Is Exposed on the SCV

**Figure 1:**
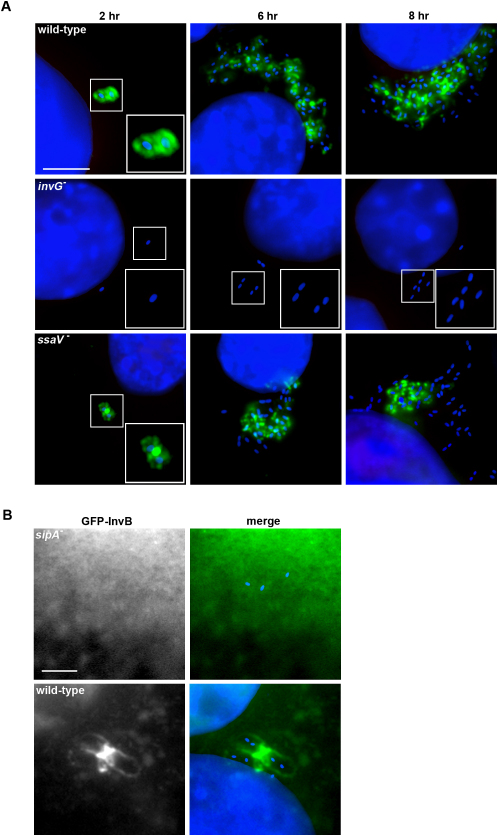
SipA Persists after *Salmonella* Entry and Is Exposed on the Cytosolic Face of the SCV (A) Intracellular SipA^FLAG^ (green) in NIH3T3 cells after infection (hr) with wild-type *S. typhimurium* or the *ssaV^−^* mutant (blue), and in J774A.1 cells infected with the *invG^−^* mutant. SipA^FLAG^ was expressed and exported equivalently to untagged SipA, retained the ability to bind F-actin, and did not influence the export or delivery of other SPI1 effectors or bacterial entry rate. Equivalent data were obtained using HeLa and J774A.1 cells (not shown). Scale bar, 5 μm. (B) GFP-InvB distribution (green) in NIH3T3 transfectants 2 hr after infection with wild-type *S. typhimurium* or the *sipA^−^* mutant (blue). GFP-InvB colocalized with SipA^FLAG^ and did not impede replication or prevent perinuclear positioning of wild-type *S. typhimurium*. Scale bar, 3 μm.

We observed that SipA remains associated with internalized bacteria (Figure S1A in the Supplemental Data available with this article online), while other SPI1 effectors are degraded (data not shown; Kubori and Galan, 2003). However, as we initially employed a wild-type *S. typhimurium* strain expressing enhanced levels of SipA from a plasmid (*sipA^++^*; Cain et al., 2004), we next examined the localization of SipA^FLAG^ expressed from the endogenous chromosomal context. SipA^FLAG^ similarly surrounded ∼60% internalized bacteria 2 hr postinfection and after 8 hr was associated with a similar proportion of microcolonies (Figure 1A). To establish which T3SS delivers this formerly unrecognized SipA^FLAG^ pool, we equivalently engineered *S. typhimurium invG^−^* and *ssaV^−^* mutants, which respectively lack essential components of the SPI1 and SPI2 T3SSs, rendering them inactive (Crago and Koronakis, 1998; Hensel et al., 1997). While SipA^FLAG^ was never detected after infection with *S. typhimurium invG^−^*, localization using the *ssaV^−^* mutant mirrored the wild-type (Figure 1A). SipA^FLAG^ staining was unchanged following bafilomycin treatment that disrupts the SPI2 T3SS (Steele-Mortimer et al., 2000), and in SPI2 effector mutants (Figure S1B). These findings demonstrate that SipA persistence requires the SPI1 T3SS post-*Salmonella* entry.

To ascertain whether SCV-associated SipA is exposed in the host cytosol, we exploited the finding that delivered SipA can be visualized during entry using its export chaperone (InvB) fused to GFP as a cytosolic reporter (Schlumberger et al., 2005). GFP-InvB transfectants were infected with wild-type *S. typhimurium* or an isogenic *sipA^−^* mutant. GFP-InvB distributed throughout the cytosol of control cells or those infected with the *sipA^−^* mutant (Figure 1B). By contrast, GFP-InvB was recruited to SCVs post-wild-type infection (Figure 1B and Figure S1C), demonstrating that SipA is exposed on the SCV and potentially poised to engage host or bacterial targets.

### SipA Promotes Intracellular Replication and Perinuclear SCV Positioning

**Figure 2:**
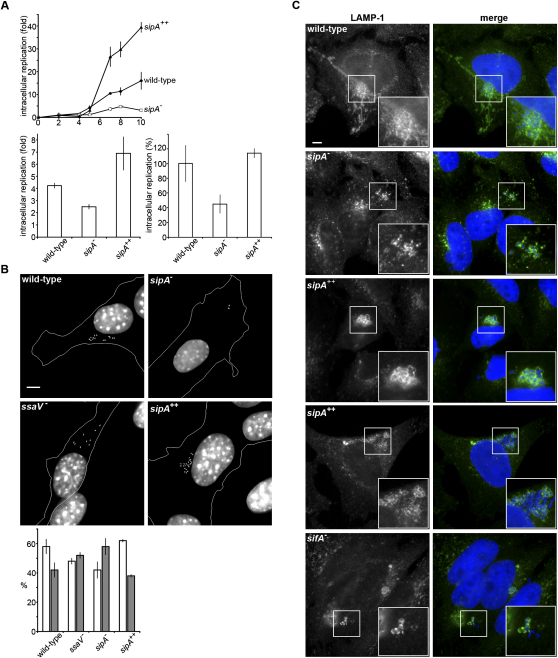
SipA Promotes *Salmonella* Replication and Is Required for SCV Positioning (A) Upper: Fold increase in intracellular wild-type *S. typhimurium* (filled circles), the *sipA^−^* mutant (*sipA^−^*, open squares), and a strain constitutively expressing augmented levels of SipA from a plasmid (*sipA^++^*, filled triangles) strain in NIH3T3 cells over time (hr). Equivalent effects were observed in HeLa cells (not shown). Lower: Fold increase (left, RAW264.7 macrophages) or percentage increase compared to wild-type (right, bone marrow-derived macrophages) of wild-type *S. typhimurium*, the *sipA^−^* mutant, and the *sipA^++^* strain over 22 hr. Replication as fold increase in intracellular bacteria was calculated by comparing values at 2 hr and subsequent time points postinfection. NIH3T3 cells were lysed after ∼11 hr due to bacterial replication. Data were derived from three independent experiments and are shown as mean ± SEM. (B) Upper: Typical distribution of wild-type *S. typhimurium*, the *sipA^−^* and *ssaV^−^* mutants, and the *sipA^++^* strain (gray) 6 hr after infection of NIH3T3 cells. Lower: The percentage of intracellular bacteria from 50 infected cells proximal (within 3 μm, open bars) and distal (>3 μm, filled bars) to the nearest edge of the nucleus 6 hr postinfection. Positioning and replication of the *sipA^−^* strain was rescued by complementation with a low-copy-number plasmid encoding *sipA* (not shown). Data were derived from three independent experiments and are shown as mean ± SEM. (C) LAMP1 (green) in HeLa cells 6 hr after infection with wild-type *S. typhimurium*, the *sipA^−^* mutant, or the *sipA^++^* or *sifA^−^* mutant strains (blue). Scale bar, 5 μm.

We next investigated whether SipA influences intracellular multiplication. Replication of the *sipA^−^* mutant was significantly attenuated, whereas that of the *sipA^++^* strain was reciprocally enhanced in epithelial-like cells and fibroblasts (Figure 2A). This dose-dependent response shows that SipA is central to *Salmonella* multiplication in nonphagocytic cells. As SPI2 effectors also govern replication in macrophages, we analyzed any comparable role for SipA using cultured and primary macrophages. Unexpectedly, the *sipA^−^* mutant was again significantly attenuated, while increasing SipA levels bolstered bacterial replication (Figure 2A). These surprising findings illustrate that SipA also influences replication in macrophages.

Attenuated *sipA^−^* mutants adopt a dispersed intracellular distribution biased toward the cell periphery (Figure 2B), reminiscent of bacteria lacking SPI2 effectors that direct SCV-organelle tethering or impede host motor protein activity (Boucrot et al., 2005; Henry et al., 2006; Salcedo and Holden, 2003). To evaluate “scattering,” every bacterium was categorized as nuclear proximal or distal. The proximal zone typically encompasses the GA (Figure S2A). Concomitant with the onset of the replicative defect, wild-type and *sipA^++^* strains appeared predominantly perinuclear, whereas *ssaV^−^* and *sipA^−^* mutants were dispersed (Figure 2B). To assess whether scattering reflected increased SCV-GA uncoupling (Abrahams et al., 2006; Salcedo and Holden, 2003), infected cells were treated with brefeldin A (BFA) that induces *cis-*Golgi redistribution into the endoplasmic reticulum (Chardin and McCormick, 1999; Lippincott-Schwartz et al., 1990). While BFA reduced wild-type and *sipA^++^* replication, the *sipA^−^* mutant was not additionally attenuated, and BFA induced scattering of both wild-type and *sipA^++^* strains, whereas positioning of the *sipA^−^* mutant was unchanged (Figure S2B). Indeed, the *sipA^−^* mutant is seldom coincident with the *cis*-Golgi (Figure S2A). Taken together, these findings implicate SipA as a positioning determinant upstream of GA association.

### Bacteria Lacking SipA Reside within Intact SCVs

Scattering and replicative attenuation of the *sipA^−^* mutant might reflect bacterial release into the host cytosol. To assess SCV integrity, LAMP1 distribution was examined in epithelial cells and fibroblasts infected with wild-type *S. typhimurium*, *sipA^−^*, and *sipA^++^* strains. Bacteria lacking the SPI2 effector SifA that are released into the host cytosol at late time points were also examined (Beuzon et al., 2000). In each infected cell, multiple wild-type bacteria were observed within continuous perinuclear SCVs, from which Sifs extended (Figure 2C and Figure S3; Movie S1). SCVs formed by the *sipA^−^* mutant remained intact but always unusually encapsulated only individual or sometimes pairs of bacteria (Figure 2C and Figure S3; Movie S2). Furthermore, Sifs rarely extended from these SCVs, although occasional “stunted” protrusions were evident (<5% SCVs). Even more unexpectedly, the *sipA^++^* strain formed continuous perinuclear SCVs that lacked Sifs, in which the membrane tightly apposed encapsulated bacteria. These tight SCVs seemed inherently unstable, as bacteria frequently became cytosolic (in ∼50% infected cells) (Figure 2C). By comparison, 30%–40% *sifA^−^* mutants were cytoplasmic at this time point (Figure 2C; (Boucrot et al., 2005). These findings demonstrate that SipA is not essential for SCV integrity, but that relative SipA concentration influences SCV morphology and positioning.

### SipA as a Key Determinant of SCV Positioning

**Figure 3:**
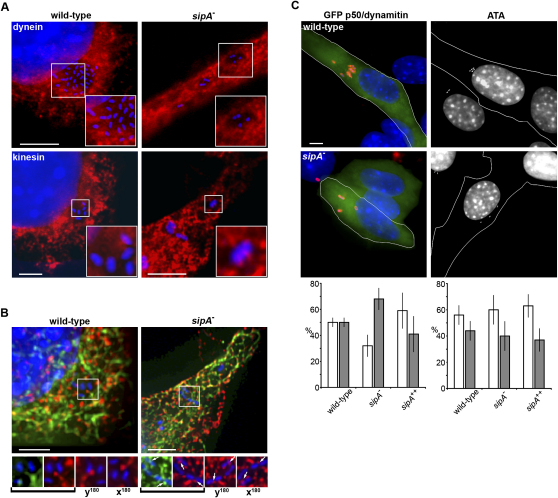
SipA Simultaneously Promotes Perinuclear SCV Migration and Prevents Kinesin Association (A) Dynein and conventional kinesin (red) in NIH3T3 cells 6 hr after infection with wild-type *S. typhimurium* or the *sipA^−^* mutant (blue). Scale bars, 3 μm (kinesin) and 5 μm (dynein). (B) Deconvolved immunofluorescence micrographs of a single z section from a rendered image showing NIH3T3 cells 6 hr postinfection with wild-type *S. typhimurium* or the *sipA^−^* mutant (blue). Scale bars, 5 μm. Colocalization between kinesin (red), tubulin (green), and the *sipA^−^* mutant is marked with arrows. Indicated regions are rotated 180° about the x (x^180^) and y (y^180^) axes. (C) Upper: Typical distribution of wild-type *S. typhimurium* and the *sipA^−^* mutant (red [left] or blue [right]) 6 hr after infection of pGFP-p50/dynamitin-transfected (left) or ATA-treated (right) NIH3T3 cells. Scale bar, 5 μm. Lower: The percentage of bacteria proximal (<3 μm, open bars) and distal (>3 μm, filled bars) to the nearest edge of the nucleus in pGFP-p50/dynamitin-transfected (left) and ATA-treated (right) NIH3T3 cells. Data were derived from three independent experiments and are shown as mean ± SEM.

*Salmonella* replication and SCV integrity require balanced activity of the microtubule motors dynein and kinesin, which respectively transport cargo toward the nucleus and cell periphery (Boucrot et al., 2005; Guignot et al., 2004; Harrison et al., 2003; Marsman et al., 2004). As SipA manipulates SCV positioning, we examined dynein and kinesin distribution in infected epithelial cells and fibroblasts. After infection with wild-type *S. typhimurium*, both dynein and kinesin accumulated diffusely around the SCV periphery but were only occasionally coincident with bacteria (Figure 3A; Boucrot et al., 2005). However, kinesin and tubulin frequently colocalized with peripheral *sipA^−^* SCVs, whereas dynein remained infrequently associated (Figure 3B).

When dynein function was disrupted with p50/dynamitin, which induces endosome redistribution to the periphery by uncoupling dynactin (data not shown; Burkhardt et al., 1997), an equivalent subtle increase (∼10%) in wild-type and *sipA^−^* at the cell periphery was observed, together with mild replicative attenuation (Figure 3C and Figure S4). These data verify that dynein contributes to perinuclear SCV positioning (Guignot et al., 2004) and demonstrate that this is SipA independent. Intriguingly, both positioning and replication of the *sipA^++^* strain are resistant to p50/dynamitin expression (Figure 3C and Figure S4). Kinesin activity was inhibited with aurintricarboxylic acid (ATA), which impeded replication of wild-type and the *sipA^++^* strain (Figure S4; Guignot et al., 2004), and although both remained perinuclear, peripheral migration of the *sipA^−^* mutant was blocked (Figure 3C). However, despite positional rescue, *sipA^−^* replication was not restored (Figure S4). Taken together, these data demonstrate that scattering of *sipA^−^* SCVs is associated with aberrant kinesin recruitment.

### SipA Modulates SPI2 Effector Localization by Binding SCV-Associated F-actin

**Figure 4:**
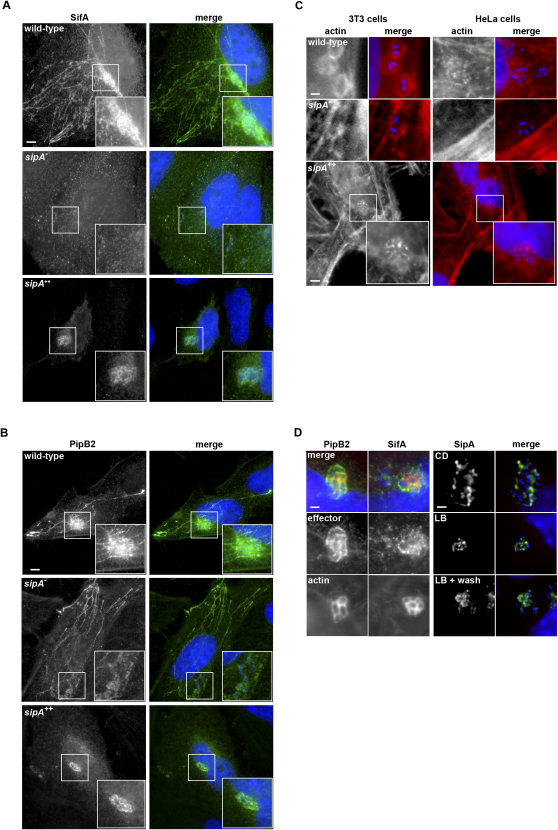
SipA Influences SPI2 Effector Localization and F-actin Stabilization around the SCV (A) SifA^HA^ localization (green) in HeLa cells 6 hr after infection with wild-type *S. typhimurium*, the *sipA^−^* mutant, or the *sipA^++^* strain (blue) expressing SifA^HA^. Scale bars, 5 μm. (B) PipB2^HA^ localization (green) in HeLa cells 6 hr after infection with wild-type *S. typhimurium*, the *sipA^−^* mutant, or the *sipA^++^* strain (blue) expressing PipB2^HA^. Scale bar, 5 μm. (C) F-actin (red) in NIH3T3 (left) and HeLa (right) cells 6 hr after infection with wild-type *S. typhimurium*, the *sipA^−^* mutant, or the *sipA^++^* strain (blue). Scale bars, 5 μm. (D) Left: Effector (green) and F-actin (red) localization 6 hr after infection of HeLa cells with the *sipA^++^* strain (blue). Scale bar, 1 μm. Right: SipA^FLAG^ (green) 6 hr postinfection of cytochalasin (CD)- or latrunculin (LB)-treated NIH3T3 cells with wild-type *S. typhimurium* (blue). Scale bar, 3 μm.

Cytoskeletal dynamics underpin endogenous vacuole biogenesis and trafficking, and both F-actin and microtubules accumulate around the SCV (Guignot et al., 2004; Meresse et al., 2001). Two SPI2 effectors influence SCV-kinesin interaction, possibly antagonistically; SifA negatively regulates kinesin activity, whereas PipB2 triggers kinesin recruitment (Boucrot et al., 2005; Henry et al., 2006). To gain further insight into the role of actin-binding SipA in SCV positioning, we examined the localization of SifA^HA^, PipB2^HA^, and F-actin after infection with wild-type, *sipA^−^*, and *sipA^++^* strains. In fibroblasts and epithelial cells, nuclear-proximal SCVs containing wild-type bacteria were enriched with SifA^HA^, which additionally decorated Sifs as expected (Figure 4A), whereas SifA^HA^ present on SCVs containing *sipA^−^* mutants was markedly reduced, and consequently Sifs seldom formed. SifA^HA^ nevertheless localized to compartments distinct from the SCV (Figure 4A). By contrast, SifA^HA^ was present but apparently dormant on “tight” Sif-devoid SCVs formed by the *sipA*^++^ strain (Figure 4A). PipB2^HA^ localized to SCVs, peripheral vesicles, and extended tubular structures after wild-type infection and remained localized with SCVs containing the *sipA^−^* mutant, consistent with its role as a kinesin linker (Figure 4B). Strikingly, as with SifA^HA^, PipB2^HA^ localized only to SCVs formed by the *sipA^++^* strain and was unable to disseminate within the infected cell (Figure 4B). These findings demonstrate that SipA imbalance induces mislocalization of SPI2 effectors SifA and PipB2, leading indirectly to aberrant positioning and morphological defects.

Given that SipA binds F-actin (Zhou et al., 1999), we next investigated any link with F-actin-SCV association, and how this might potentially impact on SifA and PipB2 localization. By 6 hr postinfection, F-actin “nests” surrounded the SCVs of wild-type bacteria, which were more evident in fibroblasts than epithelial-like cells (Figure 4C; Meresse et al., 2001). F-actin staining appeared more indistinct following equivalent infection of both cell lines with the *sipA^−^* mutant, suggesting that SipA stabilizes phagosomal F-actin (Figure 4C), akin to its role during cell entry (McGhie et al., 2004). However, F-actin was robustly enriched on the “tight” SCVs formed following infection of epithelial cells with the *sipA^++^* strain (Figure 4C). Furthermore, F-actin colocalized with both SifA^HA^ and PipB2^HA^ (Figure 4D). Excessive SCV-F-actin accumulation therefore impedes SPI2 effector activity. Nevertheless, as with SipA staining during entry (Schlumberger et al., 2005), SCV-SipA^FLAG^ association occurred when infected cells were treated with cytochalasin D (CD) or latrunculin B (LB), which prevent actin assembly and inhibit bacterial replication (Figure 4D; Meresse et al., 2001), and was unaltered even after actin assembly was reinitiated by LB washout (Figure 4D). Thus, SipA-induced F-actin stability modulates localization of key SPI2 effectors, but SipA targeting to the SCV is actin independent.

### Recognition and Centripetal Redistribution of Late Endosomes by SipA

**Figure 5:**
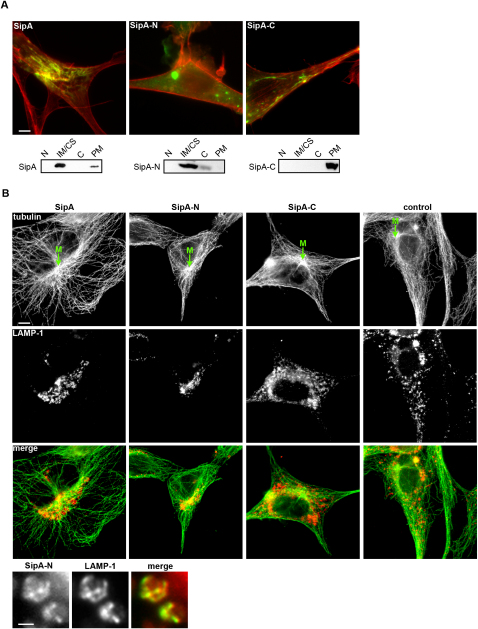
The N-Terminal Region of SipA Induces Centripetal Redistribution of Late Endosomes toward the Microtubule-Organizing Center (A) Upper: CFP-SipA, CFP-SipA-N, and CFP-SipA-C (green) NIH3T3 transfectants costained for F-actin (red). Equivalent localization was observed for comparable YFP fusions and SipA, SipA-N, and SipA-C. Scale bar, 5 μm. Lower: NIH3T3 transfectants were mechanically fractionated. Nuclear (N), internal membrane/cytoskeleton (IM/CS), cytosol (C), and plasma membrane (PM) fractions were analyzed by anti-SipA immunoblotting. (B) LAMP1 (red) and tubulin (green) localization in NIH3T3 transfectants expressing SipA, SipA-N, or SipA-C. “M” indicates microtubule-organizing center. Scale bar, 5 μm. Lower panels show perinuclear colocalization of CFP-SipA-N and LAMP1. Scale bars, 5 μm.

SipA lacks lipid affinity in vitro (Hayward and Koronakis, 1999). To delineate the region(s) of SipA involved in vacuole targeting, we transfected cells with SipA, the C-terminal actin-binding fragment (SipA-C) and the remainder that encodes no recognized activity (SipA-N) as C- or N-terminal fusions to YFP or CFP, respectively. SipA and SipA-C colocalized with F-actin, although SipA-C was more peripheral and SipA distributed throughout the cell body (Figure 5A). SipA-N fusions were never coincident with F-actin but instead exhibited punctate perinuclear distribution (Figure 5A). Fractionation revealed that, while SipA partitioned in the internal membrane/cytoskeleton fraction (Cain et al., 2004), actin-binding SipA-C was located exclusively in the plasma membrane fraction, which additionally contains ∼10% cellular actin (Cain et al., 2004), and SipA-N was predominantly in the internal membrane fraction, with ∼15% detected in the cytosolic fraction (Figure 5A).

As SipA localizes to LAMP1-rich SCVs independently of F-actin during infection, we visualized LE/Lys distribution in cells expressing SipA, SipA-N, and SipA-C. Remarkably, although neither SipA nor SipA-N trigger obvious rearrangement of the actin or microtubule networks, both induced dramatic centripetal aggregation of LAMP1-positive LE/Lys compartments toward the microtubule-organizing center (MTOC; Figure 5B). Additionally, these compartments colocalized with SipA-N (Figure 5B), demonstrating that SipA-N autonomously targets LE/Lys and induces their relocalization. Significantly, despite SipA localizing to the actin cytoskeleton, it also retained the capacity to induce similar LE/Lys redistribution, whereas actin-binding SipA-C exhibited no comparable activity.

### Coordinate Action of SipA-N and SifA Ensures Perinuclear SCV Positioning

**Figure 6:**
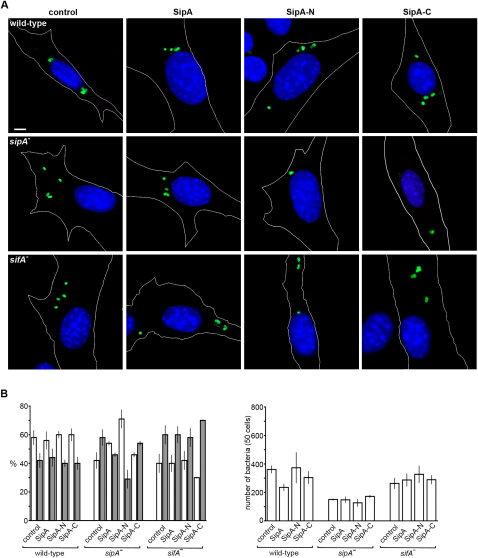
Coordinate Action of SipA-N and SifA Is Required for Perinuclear SCV Positioning (A) Typical distribution of wild-type *S. typhimurium* and the *sipA^−^* and *sifA^−^* mutants (green) 6 hr after infection of SipA, SipA-N, or SipA-C NIH3T3 transfectants. Scale bar, 5 μm. (B) Percentage of intracellular bacteria proximal (within 3 μm, open bars) and distal (>3 μm, filled bars) to the nearest edge of the nucleus (left) and the number of bacteria (right) in 50 SipA, SipA-N, or SipA-C NIH3T3 transfectants 6 hr after infection with wild-type *S. typhimurium*, and the *sipA^−^* or *sifA^−^* mutants. Data were derived from three independent experiments and are shown as mean ± SEM.

Given this previously uncharacterized ability of SipA to redistribute LE/Lys toward the nucleus, we investigated whether SipA, SipA-N, or SipA-C could complement the *sipA^−^* mutant in infected cells. Expression of SipA or either derivative had no significant effect on intracellular positioning of wild-type *S. typhimurium* (Figure 6), although SipA expression attenuated intracellular replication (Figure 6B). However, expressed SipA and SipA-N both restored perinuclear positioning to the *sipA^−^* mutant (Figure 6), with ∼75% of bacteria lacking SipA shifting into the perinuclear zone in SipA-N transfectants (Figure 6B). Yet despite these effects on positioning, expression in *trans* failed to restore replicative proficiency or Sif formation (Figure 6B; data not shown).

As SCV-localized SipA stabilizes SifA that in turn inhibits kinesin activity (Figure 4), we examined the effect of in *trans* SipA expression on intracellular replication and positioning of *sifA^−^*, *sifA^−^sipA^−^*, and control *sseI^−^* mutants. Unlike with the *sipA^−^* mutant that delivers SifA, expression of SipA, SipA-N, or SipA-C did not restore positioning or replication of the *sifA^−^* mutant (Figure 6; Figure S5). On the contrary, SipA-C expression increased peripheral positioning of the *sifA^−^* mutant. Identical results were obtained postinfection of transfectants with the double mutant lacking SipA and SifA (data not shown). These data illustrate that SipA and SifA must cooperate to ensure perinuclear SCV positioning during infection.

### SipA Dosage Is Precisely Titrated during Infection

**Figure 7:**
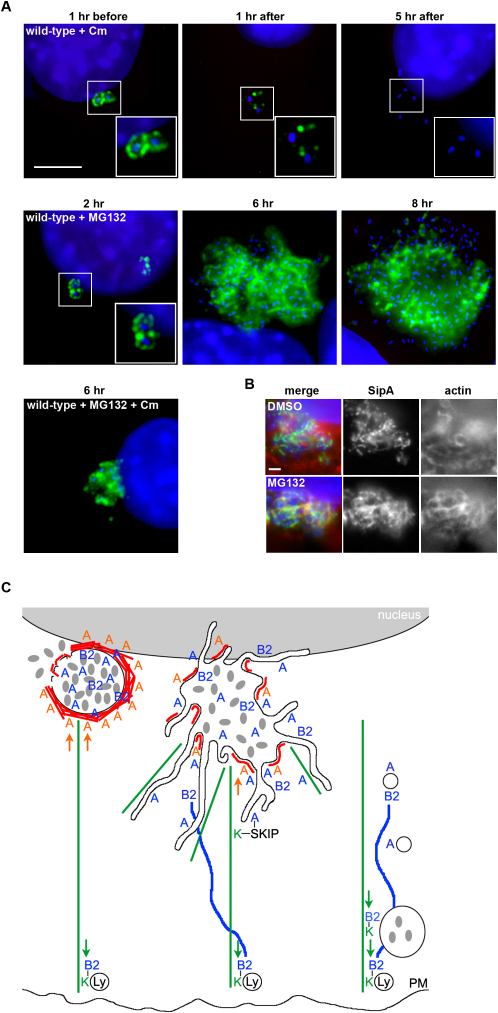
Evidence for Active Control of Intracellular SipA Concentration (A) SipA^FLAG^ (green) in NIH3T3 cells treated with chloramphenicol, MG132, or both drugs after infection (time shown in hours [hr]) with wild-type *S. typhimurium* (blue). Scale bar, 5 μm. (B) SipA^FLAG^ (green) and F-actin (red) in NIH3T3 cells treated with DMSO (top) or MG132 (bottom) 6 hr after infection with wild-type *S. typhimurium* (blue). Scale bar, 1 μm. (C) Schematic representation of the phenotypes observed after infection of nonphagocytic cells with wild-type (center), the *sipA^−^* mutant (right), and the *sipA^++^* (left) *S. typhimurium* strains. Red and green lines represent F-actin and microtubules, respectively. SPI2 effectors are shown in blue (A, SifA; B2, PipB2), SPI1 SipA (A) in orange, and host proteins SifA-kinesin interacting protein (SKIP, black) and the microtubule-dependent motor kinesin (K, green). PipB2-enriched tubular structures (blue lines) are distinct from *Salmonella*-induced filaments (Sifs).

By genetically manipulating *Salmonella*, we have demonstrated that SipA concentration profoundly influences intracellular replicative proficiency, SCV positioning, and SPI2 effector localization. We therefore predicted that SipA concentration must be tightly controlled during *Salmonella* infection. To assess this, we initially investigated the effect of inhibiting bacterial protein synthesis after entry using chloramphenicol. Only ∼15% internalized bacteria associated with SipA^FLAG^ 1 hr after antibiotic treatment, with residual immunostaining appearing fragmented and distal from the bacteria, which as expected also failed to replicate. No signal was detectable 6 hr postinfection (Figure 7A). These data show that bacterial protein synthesis is required for SipA persistence and illustrate that SipA is apparently actively degraded. Some delivered SPI1 effectors are differentially targeted by the cellular proteasome (Kubori and Galan, 2003). To confirm whether intracellular SipA is similarly susceptible to host-mediated degradation, infected cells were treated with a proteasome inhibitor (MG132). Although MG132 promoted cytosolic *Salmonella* replication (data not shown; Perrin et al., 2004), SipA^FLAG^ accumulation increased significantly (Figure 7A). This was not a function of increased bacterial load, as a pool of delivered SipA^FLAG^ could be captured following treatment with chloramphenicol and MG132 (Figure 7A). Intriguingly, inhibiting proteasome activity induced increased F-actin accumulation around the SCV and vacuole instability (Figure 7A). This phenotype mirrors that following infection with the *sipA^++^* strain, which suggests that aberrant F-actin accumulation triggers SCV instability.

## Discussion

Previous studies of SipA have detailed how the C-terminal actin-binding domain enhances bacterial entry by promoting actin polymerization and stabilizing the generated filament architecture (McGhie et al., 2001, 2004; Zhou et al., 1999). We show that SipA remains after bacterial uptake and is exposed on the cytoplasmic face of the SCV. SipA-dependent stabilization of SCV-associated F-actin is an important checkpoint during niche biogenesis, and consequently SipA must be precisely dosed. Actin binding is dispensable for SCV and LE/Lys targeting, which is directed by the previously anonymous N-terminal region. Not only is SipA-N sufficient to induce centripetal LE/Lys redistribution, but it also cooperates with the SPI2 effector SifA during infection to promote SCV trafficking toward the nucleus. Concurrently, it prevents detrimental SCV-kinesin association by localizing SifA. These combined activities ensure perinuclear SCV positioning and proficient intracellular bacterial replication.

Our finding that SipA persists after *Salmonella* entry reinforces the view that SPI1 effectors not only trigger bacterial uptake but also remain active later during infection. Our data demonstrating functional cooperativity between effectors delivered by separate T3SSs hints at an additional level of unexpected complexity. Invasion by *S. typhimurium sipA^−^* and *sopB^−^* mutants is only mildly attenuated (Zhou et al., 1999, 2001), as concerted SPI1 activities trigger entry-associated actin reorganization (Hayward and Koronakis, 2002). However, both these mutants exhibit stronger defects in replication in both epithelial cells and macrophages (Figure 2; Hernandez et al., 2004), suggesting that SipA and SopB fulfill significant roles later during infection. The contribution of individual SPI1 effectors to virulence beyond initial invasion in animals is yet to be comprehensively investigated, although SipA is also required for proinflammatory responses in epithelial cells (Lee et al., 2000) and together with other SPI1 effectors for diarrhea in cattle (Zhang et al., 2002). In agreement with our findings, the original description of the *sipA^−^* phenotype indicated an unexplained but reproducible increase in the mean time to death in mice (Kaniga et al., 1995), more indicative of a systemic replicative defect rather than a significant early invasive attenuation.

Intracellular SipA concentration must be precisely balanced to ensure a compromise between bacterial replication and cell viability. In support of this view, we observed that SipA overtitration induces accumulation of SCV-associated F-actin, leading to SPI2 effector mislocalization, SCV instability, and eventually unchecked bacterial replication in the host cytosol. Conversely, lack of SipA leads to SifA mislocalization, aberrant kinesin-dependent SCV trafficking to the cell periphery, and replicative attenuation (Figure 7C). This implies that during wild-type infection controlled stabilization of SCV-associated F-actin is a critical checkpoint that perhaps signals correct SCV positioning, priming SPI2 effector deployment. Similar factors might operate in physiological cellular trafficking pathways, where some endosomes analogously assemble tightly knit actin “coats,” but it remains unclear whether this acts to limit or selectively stimulate compartment fusion and docking events (Defacque et al., 2000; Kjeken et al., 2004; Yam and Theriot, 2004).

While both SipA and SipA-N relocate LE/Lys in uninfected cells, SipA remains additionally colocalized with F-actin. This might indicate that SipA acts in *trans* on LE/Lys while bound to F-actin or alternatively reflect that SipA has a higher affinity for actin than LE/Lys or an unknown LE/Lys-localized target in the absence of regulatory signals. This raises the possibility that during infection SipA activities are differentially controlled by an additional host factor or bacterial effector on the SCV, possibly SifA. The context of SipA activity also seems critical, as cell-expressed SipA impedes bacterial replication but can nevertheless restore SCV positioning in *trans*, whereas only bacterial expression of SipA restores both positioning and replication.

In contrast to SifA, which autonomously tubulates LE/Lys (Brumell et al., 2001), SipA and SipA-N redistribute LAMP1-positive compartments toward the MTOC without influencing their morphology, an activity more reminiscent of eukaryotic tethering factors. These cellular proteins remain poorly characterized, but some capture vesicles distal from target organelles and funnel them toward the cell body, while others, like golgins, act as organelle anchors (Behnia and Munro, 2005). Such a role is consistent with the “SCV-blind” phenotype observed with the *sipA^−^* mutant, where SifA localizes to distal compartments that fail to locate or fuse with the SCV. This would suggest that SCV-localized SipA captures SifA-positive compartments and funnels them toward the replicative compartment. Although database searches failed to detect obvious similarities, attempts using constrained SipA segments revealed that residues 121–175 share primary sequence similarity to cellular tethering factors like restin and golgins, and multiple Rab- and microtubule-interacting proteins (Figure S6). Although these observations should not be overemphasized, the crystal structure of an N-terminal SipA fragment in complex with InvB fortuitously included this region (Lilic et al., 2006). Consistent with GFP-InvB binding failing to impede SipA function, residues 121–175 appear remote from the chaperone binding domain and exposed on the opposite face of this predominantly helical region, indicating that this apparent homology might reflect a conserved interactive interface (Figure S6).

Our data reveal that *Salmonella sipA^−^* and *sifA^−^* mutants share surprisingly similar phenotypes, although there are also significant differences. SipA is required for SifA localization to the SCV, but not vice versa, whereas excess SipA induces SifA mislocalization. SipA likely excludes kinesin indirectly by localizing SifA to the SCV and/or promoting fusion of SifA-positive compartments with the SCV. In turn, SifA binds SKIP, a negative kinesin regulator (Boucrot et al., 2005). The kinesin linker PipB2 localizes to SCVs generated by bacteria lacking SipA and in the absence of SifA likely directs their migration to the cell periphery (Figure 7C; Henry et al., 2006). Cellular Rab-interacting proteins frequently stabilize their cognate GTPase and influence motor protein activity by direct binding. However, SipA is absent from Sifs, and SCV-associated SifA and SipA are not coincident. No direct interaction can be detected in vitro or between SipA-N and cellular proteins in extracts or upon yeast two-hybrid screening (our unpublished data). This suggests that SipA might be a component of a proposed multiprotein SCV-associated regulatory complex that may include SifA, SKIP, and as yet unidentified factors (Boucrot et al., 2005). Nevertheless, by priming the SCV for subsequent SPI2 effector activity, SipA provides functional continuity between forced bacterial entry and the intracellular replicative niche. Our observations open up new insights into SCV dynamics and further highlight the complexity of crosstalk between bacterial pathogens and their hosts.

## Experimental Procedures

### Bacterial Strains, Plasmids, and Mammalian Cell Culture

Bacterial strains and plasmid construction are described fully in the Supplemental Data. Bacteria were maintained on Luria-Bertani agar or cultured in tryptone-yeast (TY) medium supplemented with 10 μgml*^−^*^1^ tetracycline, 50 μgml*^−^*^1^ kanamycin, 8 μgml*^−^*^1^ chloramphenicol, or 50 μgml*^−^*^1^ ampicillin.

Mammalian cells were cultured in Dulbecco's modified Eagle's medium (DMEM) supplemented with 10% (v/v) fetal calf serum (FCS), L-glutamine, and antibiotics (Sigma). Bone marrow-derived macrophages were cultured from mouse bone marrow in medium containing 20 ngml*^−^*^1^ recombinant macrophage colony-stimulating factor (see the Supplemental Data). Cells were incubated at 37°C, 5% CO_2_.

Drug stock solutions in dimethyl sulphoxide or ethanol (Cm) were diluted at least 1:1000 in DMEM to working concentrations: 100 μgml*^−^*^1^ Cm, 5 μgml*^−^*^1^ BFA, 1 μM bafilomycin A1, 10 μM ATA and MG132, 1 μgml*^−^*^1^ CD and LB. Cells were pretreated with ATA for 3 hr, MG132 and BafA were added 30 min prior to infection, Cm and BFA were added 1 hr postinfection, and CD and LB were added 3 hr postinvasion.

### Invasion and Replication Assays

Infection of NIH3T3 and HeLa cells with *S. typhimurium* was performed as described (Garner et al., 2002). Macrophages were infected with opsonized bacteria as described (Beuzon et al., 2000).

### Transient Transfection of Cultured Cells

NIH3T3 cells were transfected using Lipofectamine, according to the manufacturer's instructions (Invitrogen).

### Immunofluorescence Microscopy

Samples were paraformaldehyde fixed, permeabilized in 0.2% Triton X-100, incubated with appropriate primary and secondary antibodies, and analyzed using a fluorescence microscope (Leica DM IRBE), as fully detailed in the Supplemental Data.

### Mechanical Fractionation of Cultured Cells

Fractionation was performed as described (Cain et al., 2004).

## Supplemental Data

Document S1. Supplemental Experimental Procedures and Six Supplemental Figures

Movie 1. SCV Formed by Wild-Type *S. typhimurium* Three-dimensional reconstruction of an SCV formed by wild-type *S. typhimurium* rendered from a z series of deconvolved immunofluorescence images. LAMP1 is shown in red, and cell nuclei and bacteria are shown in blue.

Movie 2. SCVs Formed by *S. typhimurium* Lacking SipA Three-dimensional reconstruction of SCVs formed by the *S. typhimurium sipA^−^* mutant rendered from a z series of deconvolved immunofluorescence images. LAMP1 is shown in red, and cell nuclei and bacteria are shown in blue.
