# Supplementary material for: Salmonella SPI1 Effector SipA Persists after Entry and Cooperates with a SPI2 Effector to Regulate Phagosome Maturation and Intracellular Replication
Source: Cell Host Microbe. 2007 Mar 15;1(1):63–75. doi: 10.1016/j.chom.2007.02.001 (PMC1885946; doi:10.1016/j.chom.2007.02.001)

Cell Host & Microbe, Volume *1*

Supplemental Data

Salmonella SPI1 Effector SipA Persists after Entry and Co-Operates with a SPI2 Effector to Regulate Phagosome Maturation and Intracellular Replication

Lyndsey C. Brawn, Richard D. Hayward, and Vassilis Koronakis

Supplemental Experimental Procedures

Bacterial Strains and Plasmids

S.typhimurium wild-type ATCC 14028 and isogenic derivate null mutant strains were used. *sipA*$^-$ and *ssaV*$^-$ null mutants were gifts from Samuel Miller and David Holden, respectively, whereas *invG*$^-$*, sifA*$^-$*, sseI*$^-$ and *sspH2*$^-$ null and the *sipA*$^-$*sifA*$^-$ double null mutants were constructed using Lambda red recombination (Datsenko and Wanner, 2000). Chromosomally encoded SipA was fused to a 3xFLAG tag at the C-terminus by gene replacement to facilitate immunofluorescence detection of SipA expressed at wild-type levels. Although the SipA export signal is proposed to reside at the extreme N-terminus, secretion was unexpectedly compromised when SipA derivatives containing point mutations between residues 121 and 175 or that lacked the C-terminal actin-binding domain were expressed.

Bacterial expression plasmid pB:SipA has been described (Cain et al., 2004). Briefly the *sipA* gene was cloned into pTrc99A-FF4, to allow low-level expression downstream of the Trc promoter. To generate *S.typhimurium sipA*$^{++}$ that delivers augmented levels of SipA in a wild-type background, *S.typhimurium* 14028 was transformed with pB:SipA. *S.typhimurium sipA*$^{++}$ expresses 2- and secretes 1.5-fold wild-type levels of SipA, without compromising the expression and secretion of other SPI1 or SPI2 effectors (Cain et al., 2004). Mammalian expression vector pGFP-InvB was constructed by PCR amplification of *invB* from the *S.typhimurium* 14028 chromosome and cloning the product into pEGFP-C1 (BD Biosciences). Further PCR products encoding SipA, SipA-$N_{1\text{-}458}$ and SipA-$C_{459\text{-}685}$ were cloned into pdECFP (Simpson et al., 2000) using Gateway™ technology (Invitrogen) and into pcDNA3.1(-) (Invitrogen) using standard protocols. *sifA* and *pipB2* were HA tagged as described by Brumell et al., 2002 and Knodler et al., 2003. *sifA*-2HA was cloned into pBBR1MCS2 (Kovach et al., 1995), whilst *pipB2*-2HA was cloned into pBR322 and pBBR1MCS2. GFP p50/dynamitin vector was a gift from Trina Schroer.

Mammalian Cell Culture

Mammalian cell lines used were NIH3T3 mouse fibroblasts (ATCC-CRL-1658), HeLa cells (ATCC-CCL-2), RAW 264.7 cells (ATCC-TIB-71) and J774A.1 cells (ECACC 91051511). Bone marrow derived macrophages were cultured in RPMI 1640 (Invitrogen) medium containing 10% FCS, 5% horse serum, 2mM L-glutamine, 0.05mM 2-mercaptoethanol, 10μgml^{-1} gentamicin and 1mM sodium pyruvate. Prior to use, a representative sample of bone marrow derived cells were co-stained with phycoerythrin (PE) anti-mouse F4/80 (pan macrophage marker) and CD11b-FITC (integrin α_M) and analysed by FACS. Cells were also stained with 7-AAD (7-amino-actinomycin D) to assess viability. >90% viable cells possessed both macrophage markers.

Replication and Invasion Assays in Nonphagocytic Cells

The ability of *Salmonella* strains to invade and replicate within cultured cells was assessed by gentamicin protection. *S.typhimurium* stationary phase cultures were diluted 1:500 in TY medium and incubated to maximize invasion efficiency (6 h, 37°C, 225 rpm). Washed bacteria were added at a multiplicity of infection (MOI) 100 to serum-starved cells in DMEM supplemented with L-glutamine. After incubation (37°C, 5% CO_2, 60 min), cells were

repeatedly washed with phosphate-buffered saline (PBS) and extracellular bacteria killed by the addition of DMEM supplemented with 100μgml^{-1} gentamicin, L-glutamine and 10% FCS (v/v) (37°C, 5% CO_2, 60 min). Cells were washed again with PBS and incubated further (DMEM containing 10% FCS, L-glutamine and 7μgml^{-1} gentamicin), or lysed in 10mM Tris-Cl, pH 7.4, 0.5% (v/v) Triton X-100. Serial cell lysate dilutions were plated onto LB agar.

Replication Assays in Macrophages

Macrophages were seeded into 24-well tissue culture plates at a density of 4 x 10^5 cells per well 24 hours before use. *Salmonella* were cultured at 37°C, 225 rpm until they reached an OD_{600} of 2.0, diluted 1:2 and opsonized in DMEM/RPMI 1640 containing FCS and 10% normal mouse serum at 37°C for 20 min. Bacteria were added to the macrophages at a MOI of ~100, centrifuged at 500rpm for 5 min at room temperature and incubated for a further 25 min at 37°C in 5% CO_2. The macrophages were washed once with DMEM/RPMI 1640 containing FCS and 100μgml^{-1} gentamicin and incubated in this medium for 1 h. The medium was replaced with DMEM/RPMI 1640 containing FCS and 16μgml^{-1} gentamicin and incubated in this medium for the remainder of the experiment. Cells were lysed and plated as previously described.

Infection of Transfected Cells

GFP-InvB and GFP p50/dynamitin transfectants were infected 24 h after transfection. Transfectants expressing SipA, SipA-N and SipA-C were seeded onto coverslips 24 h post-transfection and selected using 500μgml^{-1} geneticin in growth medium. Cells were grown to 70-80% confluency prior to *S.typhimurium* infection. Expression of these constructs did not adversely affect cell number or viability.

Immunofluorescence Microscopy

Samples were fixed in 3.7% paraformaldehyde (20 min, RT). Cells were then permeabilised with 0.2% (v/v) Triton X-100 in PBS (PBST; 10 min, RT), blocked in 3% (w/v) bovine serum albumin (BSA) in PBS (1h, RT), and incubated in PBST (1h, RT) with appropriately diluted antibodies. Samples were washed in PBS and sequentially incubated with AlexaFluor 488/594-conjugated secondary antibodies diluted in PBS according to manufacturer's instructions (Invitrogen). Texas Red-conjugated phalloidin (Invitrogen) and 4,6-Diamino-2-phenylindole (DAPI, Invitrogen) were used to visualise F-actin and for bacterial and nuclear staining, respectively. Samples were mounted using ProLong antifade reagent (Invitrogen) and visualised using a fluorescence microscope (Leica DM IRBE). Images were captured using a CCD digital camera (Hamamatsu) and Openlab software (Improvision), and assembled using Photoshop CS (Adobe). In some cases, low levels of bacterial DAPI staining were digitally enhanced. Three-dimensional images were deconvolved and rendered from acquired fluorescence z-sections using Volocity software (Improvision).

Antibodies and Drugs

Primary antibodies used for immunofluorescence were mouse monoclonal anti-FLAG M2 (Sigma), anti-dynein (Chemicon International), anti-HA (HA.11, Covance), anti-kinesin (Abcam), anti-GM130 (BD Biosciences), anti-LAMP1 H4A3 (Developmental Studies Hybridoma Bank, Iowa), sheep polyclonal anti-tubulin (Cytoskeleton Inc), rabbit polyclonal anti-SipA IgG (McGhie *et al*., 2001), rabbit polyclonal anti-*Salmonella* (Biodesign International) and rat anti-CD107a (BD Biosciences).

Cm, BFA, bafilomycin A1, ATA, CD and LB were purchased from Sigma and MG132 from Calbiochem. ATA and MG132 do not affect *S.typhimurium* viability or invasion rate, and unless otherwise stated, agents were retained throughout subsequent incubation and washing steps.

Mechanical Fractionation of Cultured Cells

Transfected NIH3T3 cells were released from flasks by sequential incubation with 5 mM EDTA in Hanks-buffered saline solution (HBSS; 30 min, 37°C), then 1% (w/v) Type IV collagenase, 1% (w/v) bovine serum albumin, 1mM $CaCl_2$ in HBSS (10 min, 37°C). Cells were washed with buffer A [20mM 2-[*N*-Morpholino]ethanesulphonic acid (MES) pH 5.0, 150mM NaCl, 280 mM sorbitol]. Pelleted cells were resuspended in 1 vol buffer A and mixed with 5 vol 1% (v/v) colloidal silica in buffer A (EKA Chemicals). Unbound silica was removed by washing with buffer A. Cells were resuspended again in 1 vol buffer A and rapidly mixed with 5 vol 200 μgml^{-1} polyacrylic acid (PAA) in buffer A, pH 5.0. Excess PAA was removed by washing with buffer A, and cells resuspended in 5 vol chilled buffer B [2.5 mM imidazole, pH 7.0, Complete™ protease inhibitor cocktail (Roche)] and incubated (30 min 4°C). Samples were passed through a dounce homogenizer (HGM Precision Engineering; 8.02 mm bore, 6μm clearance),

and lysates clarified (900 *g*, 10 min), generating two fractions, the supernatant (cell cytosol, cytoskeleton and internal membranes) and the pellet (nuclei, silica-coated plasma membranes). The supernatant was sub-fractionated by centrifugation (180 000 *g*, 60 min), yielding two fractions, the supernatant (cell cytosol) and pellet (internal membranes and cytoskeleton). The original pellet was subsequently resuspended in 20 vol buffer B, layered onto 5 vol Optiprep™ (Alexisshield, 60% (w/v) Iodixanol in water) and centrifuged (27 000 *g*, 30 min). Pelleted material (plasma membrane) was separated from material at the cushion interface (nuclei). Proteins were precipitated with 10% (v/v) TCA, resuspended in SDS loading buffer and analysed by SDS-PAGE and immunoblotting. Fractions were probed with diagnostic cellular markers as previously (Cain et al., 2004).

Supplemental References

Brumell, J. H., Goosney, D. L., and Finlay, B. B. (2002). SifA, a type III secreted effector of Salmonella typhimurium, directs Salmonella-induced filament (Sif) formation along microtubules. Traffic *3*, 407-415.

Cain, R. J., Hayward, R. D., and Koronakis, V. (2004). The target cell plasma membrane is a critical interface for Salmonella cell entry effector-host interplay. Mol Microbiol *54*, 887-904.

Datsenko, K. A., and Wanner, B. L. (2000). One-step inactivation of chromosomal genes in Escherichia coli K-12 using PCR products. Proc Natl Acad Sci U S A *97*, 6640-6645.

Knodler, L. A., Vallance, B. A., Hensel, M., Jackel, D., Finlay, B. B., and Steele-Mortimer, O. (2003). Salmonella type III effectors PipB and PipB2 are targeted to detergent-resistant microdomains on internal host cell membranes. Mol Microbiol *49*, 685-704.

Kovach, M. E., Elzer, P. H., Hill, D. S., Robertson, G. T., Farris, M. A., Roop, R. M. 2nd, and Peterson, K. M. (1995). Four new derivatives of a broad host range cloning vector pRRE1MCS, carrying different antibiotic resistance cassettes. Gene *166,* 175-176.

Lilic, M., Vujanac, M., and Stebbins, C. E. (2006). A common structural motif in the binding of virulence factors to bacterial secretion chaperones. Mol Cell *21*, 653-664.

Simpson, J. C., Wellenreuther, R., Poustka, A., Pepperkok, R., and Wiemann, S. (2000). Systematic subcellular localization of novel proteins identified by large-scale cDNA sequencing. EMBO Rep *1*, 287-292.

Figure S1. SipA Persists after Entry and Is Exposed on the Cytosolic Face of the SCV

(A) Intracellular SipA (green) in NIH3T3 cells after infection (h) with wild-type *S.typhimurium* expressing 2-fold augmented levels of SipA from a plasmid (*sipA*$^{++}$, blue). Scale bar, 5μm.

(B) Intracellular SipA^{FLAG} (green) in NIH3T3 cells 6 h after infection with individual *S.typhimurium sifA*$^{-}$, *sseI*$^{-}$, or *sspH*$^{-}$ mutants (blue), or after bafilomycin A1-treated cells were infected with wild-type *S.typhimurium* (blue). Each strain was engineered to express SipA^{FLAG}. Scale bar, 5μm.

(C) GFP-InvB distribution (green) in NIH3T3 transfectants 6 h after infection with wild-type *S.typhimurium* (red). Scale bar, 3μm.

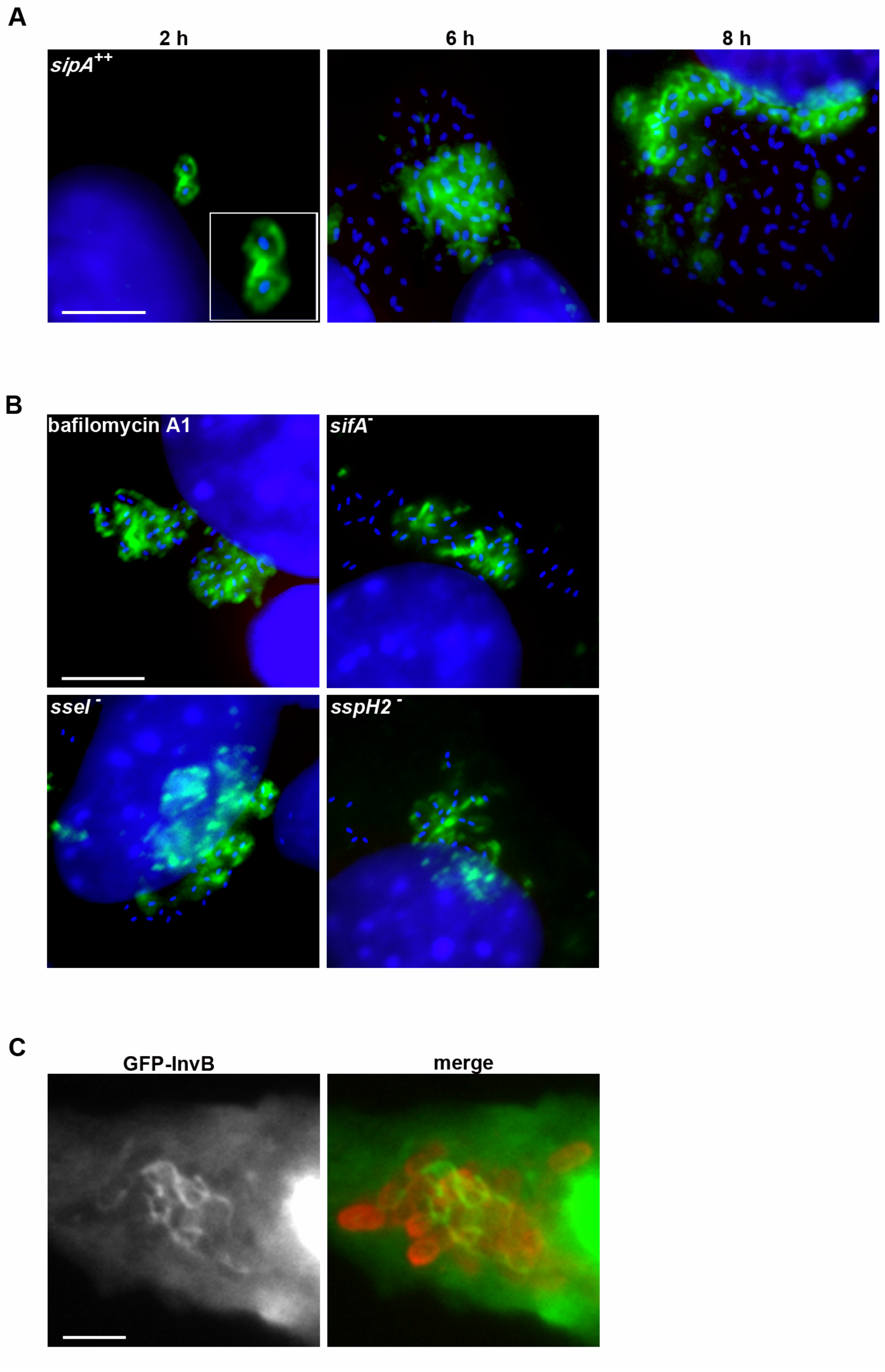

Figure S2. SipA Acts on the SCV Prior to Golgi Targeting

(A) Localization of wild-type *S. typhimurium*, the *sipA*⁻ mutant or the *sipA*++ strain (blue) in comparison to the *cis*-Golgi (green) in NIH3T3 cells 6 h post-infection. Scale bar, 5μm.

(B) Typical distribution of wild-type *S.typhimurium* and the *sipA*⁻ mutant (grey) 6 h after infection of brefeldin A-treated NIH3T3 cells. Scale bar, 5μm. The number of intracellular bacteria 6 h after infection of 50 brefeldin A-treated cells (left), and the percentage of these bacteria proximal (< 3μm, open bars) and distal (> 3μm, filled bars) to the nearest edge of the nucleus (right). Data derive from three independent experiments and are shown as mean±SEM.

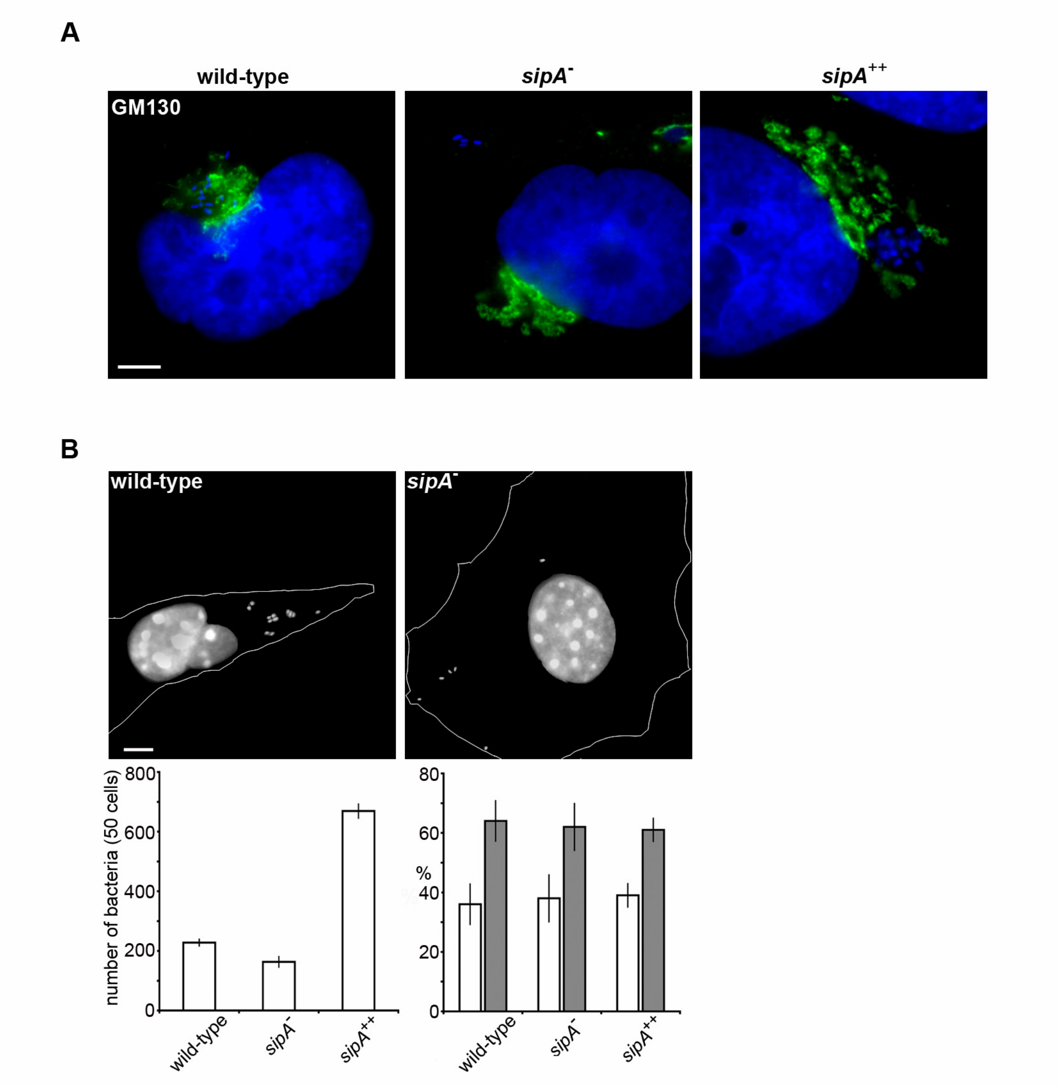

Figure S3. Bacteria Lacking SipA Reside within an Intact SCV in Fibroblasts

(A) LAMP1 (red) in NIH3T3 cells 6 h after infection with wild-type *S.typhimurium*, the *sipA*$^-$ or *sifA*$^-$ mutants (blue). Scale bar, 3 µm.

(B) Deconvolved immunofluorescence micrographs showing LAMP1 (red) in NIH3T3 cells 6 h after infection with wild-type *S.typhimurium* or the *sipA*$^-$ mutant (blue). Scale bar, 5 µm.

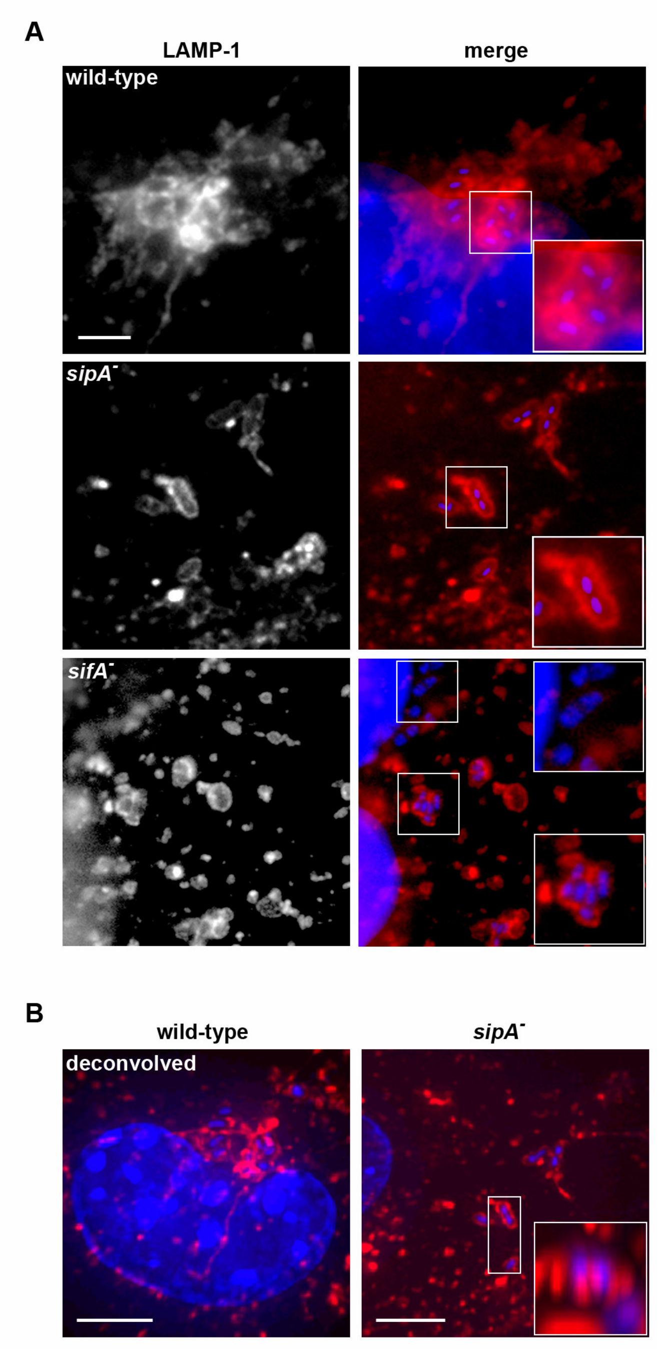

Figure S4. Inhibition of Microtubule Motors Reduces *Salmonella* Replication

Number of intracellular bacteria 6 h after infection of GFP-p50/dynamitin transfected (left) or ATA treated (right) NIH3T3 cells. Data derive from three independent experiments and are shown as mean±SEM.

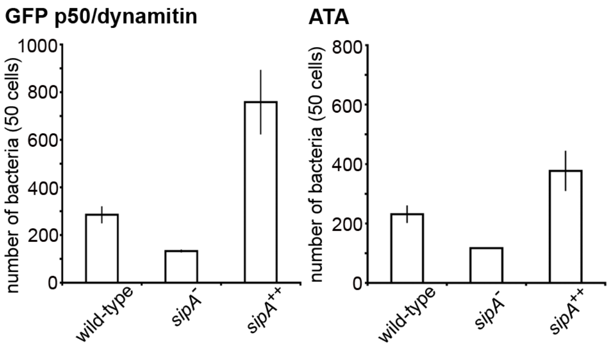

Figure S5. Coordinate Action of SipA-N and SifA Is Required for Perinuclear SCV Positioning

Percentage of intracellular bacteria proximal (within 3μm, open bars) and distal (> 3μm, filled bars) to the nearest edge of the nucleus (upper) and the number of bacteria (lower) in 50 SipA, SipA-N and SipA-C NIH3T3 transfectants 6 h after infection with *S.typhimurium ssaV⁻, sseI⁻* or *sifA⁻* mutants. Data derive from three independent experiments and are shown as mean±SEM.

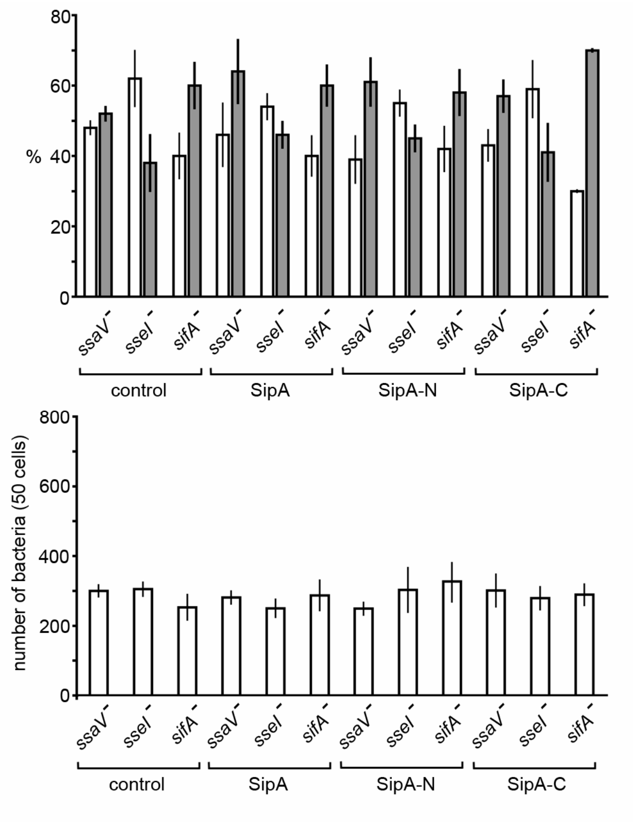

Figure S6. Primary Sequence Similarity between SipA and Cellular Proteins Involved in Intracellular Trafficking

Upper, Sequence alignment comparing SipA residues 121-180 and eukaryotic proteins derived from BLAST database searches using the BLOSUM62 matrix (existence 11; extension 1). Numbers in brackets denote multiple hits between the index and target proteins. Invariant and similar residues are highlighted in red and orange, respectively. Primary sequence identity is >35%. Lower, left, Region of sequence homology mapped onto the SipA 48-264 crystal structure (Lilic et al., 2006). Residues 121-180 are shown in blue, with amino acids identical to *H.sapiens* restin (CLIP-170) in red with side-chains. Right, 'Rear view' image, in which the left image is rotated 180º about the vertical axis. Consistent with our finding that GFP-InvB binding failed to impede SipA function, residues 121-180 are remote from the chaperone-binding domain.

```
121  AEYAAQIIKDGLKEKSAFGPWLPETKKAEAKLENLEKQLLDIIKNNTGGELSKLSTNLVM 180   SipA S.typhimurium
1077 AEDAMQIMEQMTKEKTETLASLEDTKQTNAKLQN----ELDTLKENNLKNVEELN----- 1127  Restin (CLIP-170) H.sapiens (7)
1041 AEDAMQIMEQMTKEKTETLASLEDTKQTNARLQN----ELDTLKENNLKTVEELNK---- 1092  Restin (CLIP-170) M.musculus (5)
1474 -EEELKALEDRLESESAAK--LAELKKAEQKIAAIKKQLLEQYKKGTESHLSELNTKL-- 1536  Golgin-245 H.sapiens (10)

919  ------LLKDSLEKSPSVKDQLSLVKELEEKIESLEKESKD------------------- 953   Golgi coiled-coil protein (GCC185) M.musculus (3)
927  ------LLKDSLKSPSVKNDPLSSVKELEEKIENLEKE---------------------- 959   GRIP and coiled-coil protein H.sapiens (3)
865  AEYEAQMIEDEQREKSVSHQTVQEKEQALADLNSVEKSLADLFR---------------- 915   Transforming acidic coiled-coil protein (TACC2) H.sapiens

278  --------------ETAVDPTLPKSYEAMKLSVNLKSMSASIRENNQTPELVKFSLN--- 320   Vacuolar protein sorting protein 13A G.gallus (4)
845  -----------LKGSVDFGPEVTSSDKSLKVLLNAEDKVFNEIRNNVFGFLSQKARNL-- 895   Vacuolar protein sorting protein 33B P.troglodytes (4)
243  ----SQVVYEGLVDDTFFGPEVTSSDKSLKVLLNAEDKVFNEIRNNVFGFLSQKARNL-- 308   Vacuolar protein sorting protein 33B H.sapiens (4)

256  ----------------------PRKKKSELEFEALKTPDLDVPKENITSD-TLLTTNM-- 290   Rab11 binding protein B.taurus (2)
356  --------EDGLKEKNEIIARLEETNKITAAMRQLEQRL--------------------- 387   RUN- and FYVE-domain containing protein RabIP4R H.sapiens (2)
555  -----------LKAKSALESILASERLPFSCLKNITQTLMDTLKN--------------- 588   Rab3 GTPase-activating protein B.taurus
604  -EHASSLASSGLKKDSALEQKKEECLKMESQLKKVEEKLYPLILNS-------------- 655   Rab6 interacting protein M.musculus (3)

85   -----QRLKDHLRSRSLFEQLEEKTKECEKKLESLSKET-DVLKNQLSATTKRLS----- 140   Centrosomal protein 55 M.musculus (3)
98   -----QVLLDVRRSLRRFPPGMPEEQR-----EGLQEELIDII----------------- 130   TBC1 domain family member 20 H.sapiens (2)
230  ----------------AFSKWEPDSTKKEMSLKNFVG--TDIIK---GAKSFQLIDNLL- 267   Kinetochore-associated protein 1 M.musculus (7)
1088 -------LQSGPESSSSPGVHVRQTKEAPAKLESQAGQQVDRVRSMSGG----------- 1134  Tuberous sclerosis protein 2 H.sapiens (2)
738  ----AQQLKDLLTEWETQGNQVQELNKNSSQLENL------------------------- 768   Microtubule-actin crosslinking factor D.rerio (4)

973  ---------EGLLAKALSGPFMQEQRSTEQQLGALEHQFLNILNN--------------- 1011  Dynein heavy chain M.musculus (7)
393  ----SQLLVPGASVPSPLRPWGPQTKSAKS------------------------------ 418   Synapsin-3 R.norvegicus (2)
1238 AEEVLRAHEEQLKEAQAVPATLPELEATKASLKKLRAQ---------------------- 1275  Plectin 1 H.sapiens (8)
```

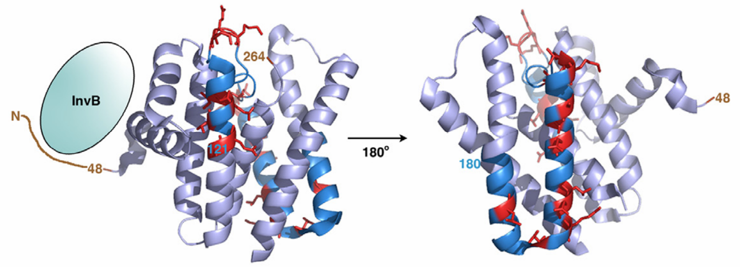

Supplement: Document S1. Supplemental Experimental Procedures and Six Supplemental Figures [file mmc1.pdf]
